# Estimating energetic intake for marine mammal bioenergetic models

**DOI:** 10.1093/conphys/coac083

**Published:** 2023-02-04

**Authors:** Cormac G Booth, Marie Guilpin, Aimee-Kate Darias-O’Hara, Janneke M Ransijn, Megan Ryder, Dave Rosen, Enrico Pirotta, Sophie Smout, Elizabeth A McHuron, Jacob Nabe-Nielsen, Daniel P Costa

**Affiliations:** SMRU Consulting, Scottish Oceans Institute, University of St Andrews, East Sands, St Andrews, KY16 8LB, UK; Department of Coastal Systems, NIOZ Royal Netherlands Institute for Sea Research, 1790 AB, Den Burg, Texel, the Netherlands; Department of Freshwater and Marine Ecology, IBED, University of Amsterdam,1090 GE, Amsterdam, the Netherlands; SMRU Consulting, Scottish Oceans Institute, University of St Andrews, East Sands, St Andrews, KY16 8LB, UK; Sea Mammal Research Unit, Scottish Oceans Institute, East Sands, University of St. Andrews, St. Andrews, KY16 8LB, UK; SMRU Consulting, Scottish Oceans Institute, University of St Andrews, East Sands, St Andrews, KY16 8LB, UK; Institute for the Oceans and Fisheries, University of British Columbia, 2202 Main Mall, Vancouver, BC V6T 1Z4, Canada; Centre for Research into Ecological and Environmental Modelling, The Observatory, Buchanan Gardens, University of St. Andrews, St. Andrews, KY16 9LZ, UK; Sea Mammal Research Unit, Scottish Oceans Institute, East Sands, University of St. Andrews, St. Andrews, KY16 8LB, UK; Cooperative Institute for Climate, Ocean, and Ecosystem Studies, University of Washington, 3737 Brooklyn Ave NE, Seattle, WA, 98105, USA; Marine Mammal Research, Department of Ecoscience, Aarhus University, Aarhus, DK-4000 Roskilde, Denmark; Ecology and Evolutionary Biology Department, University of California Santa Cruz, 130 McAlister Way, Santa Cruz, CA, 95064, USA

**Keywords:** marine mammals, energy intake, Bioenergetics

## Abstract

Bioenergetics is the study of how animals achieve energetic balance. Energetic balance results from the energetic expenditure of an individual and the energy they extract from their environment. Ingested energy depends on several extrinsic (e.g prey species, nutritional value and composition, prey density and availability) and intrinsic factors (e.g. foraging effort, success at catching prey, digestive processes and associated energy losses, and digestive capacity). While the focus in bioenergetic modelling is often on the energetic costs an animal incurs, the robust estimation of an individual’s energy intake is equally critical for producing meaningful predictions. Here, we review the components and processes that affect energy intake from ingested gross energy to biologically useful net energy (NE). The current state of knowledge of each parameter is reviewed, shedding light on research gaps to advance this field. The review highlighted that the foraging behaviour of many marine mammals is relatively well studied via biologging tags, with estimates of success rate typically assumed for most species. However, actual prey capture success rates are often only assumed, although we note studies that provide approaches for its estimation using current techniques. A comprehensive collation of the nutritional content of marine mammal prey species revealed a robust foundation from which prey quality (comprising prey species, size and energy density) can be assessed, though data remain unavailable for many prey species. Empirical information on various energy losses following ingestion of prey was unbalanced among marine mammal species, with considerably more literature available for pinnipeds. An increased understanding and accurate estimate of each of the components that comprise a species NE intake are an integral part of bioenergetics. Such models provide a key tool to investigate the effects of disturbance on marine mammals at an individual and population level and to support effective conservation and management.

## Teaser

Understanding energy balance requires robust estimates of energy intake. Bioenergetics provides a key tool to investigate the effects of disturbance in marine mammals. We review the mechanistic components that affect energy intake and the processes from ingested energy to net utilizable energy. We summarize existing datasets and highlight key research gaps to advance this field to ensure effective conservation and management.

## Introduction

Achieving energetic balance is a key to survival and reproduction in animals (Costa and Williams, 1999; Parsons, 2005; Stubbs and Tolkamp, 2006). Energetic balance results from an animal’s energetic costs (e.g. the effort expended on movement, maintenance of body processes, growth and reproduction), and the energy they can extract from their environment (Schneider, 2004). Bioenergetics, the study of how animals achieve such balance, integrates biotic and abiotic influences, including intrinsic and extrinsic factors (see Pirotta, 2022, this Special Issue), and can be used in conservation to understand the effects of stressors on an individual and the resulting dynamics on populations (Costa, 2012; Chimienti *et al*., 2020; Gallagher *et al*., 2021; Keen *et al*., 2021). Although several recent studies have advanced our understanding of the processes that influence energy use in animals, relatively little is known of the trade-offs that control energy intake. Mammalian species exhibit a wide range of life-history strategies, from long-lived species with long inter-birth intervals to species reaching sexual maturity early and with high reproductive output. Quantifying energy balance is critical to understand a species’ biology, the effect of a changing ecosystem on a species and informing effective conservation measures.

The past 40 years have seen significant advances in bioenergetic models to achieve a variety of research and conservation objectives for marine mammals. A key concern is that anthropogenic disturbance can cause behavioural, physiological and health changes that can affect an individual’s vital rates, such as survival and reproduction (Nabe-Nielsen *et al*., 2018; Pirotta *et al*., 2018a). The probability and effects of disturbance are ultimately mediated by the state of the individual (e.g. life history stage, exposure history) and the environment (e.g. resource availability) (Pirotta *et al*., 2018a; Keen *et al*., 2021). Globally, climate change is altering ecosystems, and assessing the effects of this and other anthropogenic stressors (and their complex interactions) remains a critical knowledge gap (National Academies of Sciences Engineering and Medicine, 2017; Hazen *et al*., 2019; Malhi *et al*., 2020; Gallagher *et al*. 2021b; Pirotta *et al*., 2022).

Energy intake is an important component of bioenergetic models (Pirotta, 2022, this Special Issue). While accounting models generally use a summary of energetic costs and efficiencies to estimate food intake requirements, dynamic models predict the mutual relationship between energy expenditure and energy intake. In this review, we compiled existing data on all aspects of energy intake relevant to bioenergetics models, ranging from prey acquisition and ingestion of food (including maximum rate of food intake, nutritional value of prey, prey density and food processing rates) to net energy (NE) (including losses associated with faecal energy [FE], urinary energy (UE) and heat increment of feeding [HIF]). This mechanistic approach to energy intake is population specific as it encompasses information about prey type, distribution and density and therefore individual foraging strategies and behaviours (McHuron *et al*., 2018; Pirotta *et al*., 2018b; Guilpin *et al*., 2019; Pirotta *et al*., 2019). However, it provides a useful framework to review each of these key parameters separately and identify data gaps to guide future efforts.

## Review Scope and Structure

**Figure 1:**
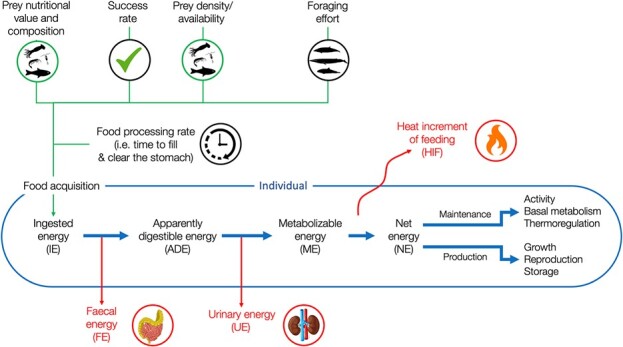
Schematic representation of the energy flow through a marine mammal (adapted from Cost a, 2009 and Lavigne et al. 1982). Green lines represent parameters influencing food acquisition an d ingested energy (IE). The outer blue line represents the individual and blue arrows represent the e nergy flow from ingested energy (IE) to net energy (NE) and subsequent allocation. Red arrows ind icate energy losses. NB: this schematic representation is not intended to represent parameters intera ctions but to provide a conceptual framework to visualize parameters and the underlying equations of a bioenergetic mechanistic approach.

This review examines the parameters and constraints used in determining the amount of energy consumed and retained by an individual. This review is novel in bioenergetics as it follows a mechanistic approach to energy intake, highlighting parameters relevant for bioenergetic models (Figure 1). We summarize the current state of knowledge regarding how acquired energy flows from ingested energy (IE) to NE. NE is subsequently used for maintenance, which includes energy used for activity, basal metabolism and/or thermoregulation and production energy, which includes growth, reproduction and storage. Energy use and allocation is addressed in other reviews of this Special Issue (growth: Adamczak *et al*., reproduction: McHuron *et al*.; metabolic rates: Noren). This review focuses on cetaceans and pinnipeds. Thus, literature on polar bears (*Ursus maritimus*), sirenians and sea otters (*Enhydra lutris*) has not been as thoroughly covered.

## Prey Acquisition

### Foraging effort and success rate

Meeting energetic requirements is dependent upon the ability of an animal to find, ingest and digest suitable prey. Generating estimates of IE begins with robust estimates of the amount of time an animal spends foraging; for marine mammals the time allocated to this activity is also constrained by having to return to the surface to breathe and/or to land to haul out (Mori, 1998; Thompson and Fedak, 2001; Rosen *et al*., 2007). Estimates of energy intake can come from functional response relationships (Mackinson *et al*., 2003), time-activity budgets (McClintock *et al*., 2013; Jeanniard-du-Dot *et al*., 2017) and estimates of prey capture attempts and their success rate (i.e. the percentage of attempts that result in the successful capture of prey) (Johnson *et al*., 2006; Wisniewska *et al*., 2016).

Biologging deployments provide information on the movement and dive behaviour of individuals and increasingly greater detail on foraging behaviour across a wide range of species (McIntyre, 2014). Such data, particularly from longer-term tags (e.g. McConnell *et al*., 1999 ; Schorr *et al*., 2017 ; Savoca *et al*., 2021), can also be used to estimate the number of typical foraging dives over defined time intervals and allow for the characterization of time-activity budgets for cetacean and pinniped species (Russell, 2015; Jeanniard-du-Dot *et al*., 2017) and how those budgets can be altered when animals are disturbed from baseline behaviour (Bejder *et al*., 2009; Isojunno *et al*., 2017). For example, Isojunno *et al*. (2016) observed that sperm whales (*Physeter macrocephalus*) spent more time in an ‘active non-foraging state’ following exposure to a low-frequency active sonar or killer whale (*Orcinus orca*) sound playback (thereby reducing time spent foraging over the tag deployment). As discussed below, these data have allowed for estimates of energy intake or balance in a variety of marine mammal species (e.g. Goldbogen *et al*., 2019; Booth, 2020; Czapanskiy *et al*., 2021; Kienle *et al*., 2022).

In pinniped species, data from non-acoustic animal-borne sensors (e.g. accelerometry and cameras) have been widely used to identify foraging events at different temporal resolutions (Viviant *et al*., 2010; Iwata *et al*., 2012; Gallon *et al*., 2013; Cole *et al*., 2021; Vance *et al*., 2021). Head movements, associated with either raptorial or suction feeding techniques, can successfully be detected using the rate of change in acceleration (or also called jerk) from sensors when adequately deployed close to the head of the individuals (Ydesen *et al*., 2014; Volpov *et al*., 2015; Cole *et al*., 2021). Experiments on captive animals or the concomitant use of camera tags on wild animals (Volpov *et al*., 2015) corroborated the use of rate of change in acceleration to identify prey-capture events.

In cetaceans, prey-capture attempts have typically been estimated using tags that collect acoustic and 3D accelerometry data (e.g. Johnson and Tyack, 2003; Lewis *et al*., 2018). For odontocetes, high-resolution acoustic biologging sensors record echolocation behaviour (e.g. the fast production of successive clicks qualified as terminal or foraging buzzes), which can be combined with associated movement characteristics (e.g. jerk) to estimate the number of prey capture attempts occurring in a dive. Foraging buzzes (with or without estimates of jerk) have been quantified in beaked whales (Family *Ziphiidae*) (Johnson *et al*., 2006; Stimpert *et al*., 2014; Siegal, 2020; Alcázar-Treviño *et al*., 2021; Visser *et al*., 2022), sperm whales (including *Kogia spp.*) (Fais *et al*., 2016; Tønnesen *et al*., 2020; Malinka *et al*., 2021), short-finned pilot whales (*Globicephala macrorhynchus*) (Aguilar Soto *et al*., 2008; Holt *et al*., 2021), narwhals (*Monodon monoceros*) (Ngô *et al*., 2021), smaller delphinids (Wisniewska *et al*., 2014; Arranz *et al*., 2016) and harbour porpoises (*Phocoena phocoena*) (Wisniewska *et al*., 2014; Wisniewska *et al*., 2018).

In mysticetes, biologging devices have been used to record a broader range of foraging strategies than observed for odontocetes, with studies of blue whales (*Balaenoptera musculus*) (Doniol-Valcroze *et al*., 2011; Goldbogen *et al*., 2011), fin whales (*Balaenoptera physalis*) (Panigada *et al*., 1999; Goldbogen *et al*., 2006), humpback whales (*Megaptera novaeangliae*) (Friedlaender *et al*., 2009, 2013; Owen *et al*., 2015; Burrows *et al*., 2016), Antarctic minke whales (*Balaenoptera bonaerensis*) (Friedlaender *et al*., 2014), Bryde’s whales (*Balaenoptera brydei*) (Alves *et al*., 2010), North Atlantic right whales (*Eubalaena glacialis*) (Baumgartner *et al*., 2003; Laidre *et al*., 2007; van der Hoop *et al*., 2019) and bowhead whales (*Balaena mysticetus*) (Simon *et al*., 2009; Heide-Jørgensen *et al*., 2013). Balaenopterids (rorquals) are bulk feeders that engulf large volumes of water and ensnared prey by expanding their ventral groves, following an abrupt acceleration phase (Croll *et al*., 2001; Goldbogen *et al*., 2006). This characteristic feeding technique called lunge feeding is associated with distinct kinematics (e.g. swim speed, overall dynamic body acceleration [ODBA], minimum specific acceleration [MSA] or rate of acceleration [jerk]), which are used to identify feeding events on dive profiles obtained from biologging devices (e.g. DTAG, TDR Wildlife computer, Acousonde, Crittercam, CATs tag, Little Leonardo). Body kinematics during foraging can inform on feeding attempts, indeed the ODBA, the rate of change in acceleration or differential of the three acceleration axes (jerk) or MSA are proxies of energy expenditure and obtained from three-dimensional accelerometry (Wilson *et al*., 2006). Although very useful, measures of ODBA can differ from small to larger marine mammals and therefore could underestimate energy expenditure in large animals (Martín López *et al*., 2022) but limitations are covered in another review on metabolic rates of this Special Issue (Noren). Nevertheless, sounds produced by odontocetes can then be used to identify potential feeding attempts. Body kinematics remains an accurate metric to identify feeding attempts (Goldbogen *et al*., 2017). Contrary to balaenopterids, balaenids engage in continuous ram filter-feeding, during which they skim through a layer of prey at low speed to reduce drag (Werth, 2004). As they do not have discrete feeding attempts, identifying foraging time within balaenid dive profiles is less straightforward and relies on the shapes of dives and fluking gaits (i.e. types) (Nowacek *et al*., 2001; Baumgartner *et al*., 2003; Laidre *et al*., 2007). One time-depth–recorder tag has been deployed on a benthic-feeding grey whale (*Eschrichtius robustus*) (Malcolm *et al*., 1996), from which dive types were classified (Malcolm and Duffus, 2000) and used to assess foraging activity in focal-follow observations (Feyrer and Duffus, 2015). In shallow intertidal habitats, benthic feeding has been confirmed from mud plumes and feeding pits (Calambokidis *et al*., 2018).

It is important to highlight that most tag deployments are short duration and thus provide only a snapshot of foraging behaviour, the representativeness of which is hard to assess. Furthermore, foraging metrics are likely to be site specific, depending on environmental, seasonal and biotic conditions. For example, blue whales tagged with DTAGs (Johnson and Tyack, 2003), VTDRs (Mk8; Wildlife Computers), National Geographic CritterCam (Marshall, 1998) and Bioacoustic Probe (B-probe; Greeneridge Sciences) in Southern California (Oleson *et al*., 2007), the St. Lawrence estuary (Doniol-Valcroze *et al*., 2011; Guilpin *et al*., 2019) and northern Chilean Patagonia (Caruso *et al*., 2021) exhibited different feeding rates. While they follow the diurnal vertical migration of their prey in each location, feeding depth and dive duration varied across locations (Oleson *et al*., 2007; Doniol-Valcroze *et al*., 2011; Guilpin *et al*., 2019; Caruso *et al*., 2021). This highlights the need to take prey density and availability into account to contextualize foraging behaviour.

Prey-capture success is likely to vary significantly across predator and prey species, depending on the foraging strategy of the predator, the morphology of the feeding apparatus, and prey density, predictability and behaviour (e.g. diurnal or nocturnal, shoaling or burring, escape strategies). Stomach temperature telemetry has been used in pinnipeds to quantify prey-capture success, as the stomach temperature recovers faster after water ingestion than after the ingestion of a prey item, although the method does not prevent false detection of prey captures (Kuhn and Costa, 2006). Additionally, distinct jerk movements and jaw movements have been identified in pinniped tag deployments, which may be used to estimate foraging success. Despite the large number of acoustic tags deployed, few studies have estimated prey-capture success rate in odontocetes. Wisniewska *et al*. (2016) estimated that harbour porpoises, fitted with DTAGs, had mean prey-capture success rates between 92–99% (*n* = 4). The prey-capture success rate has been difficult to estimate for bulk feeders, such as mysticetes. Because of the high costs associated with lunging for rorquals and the increased drag costs of skimming balaenids, it is likely that all feeding attempts would be at least partly successful (Goldbogen *et al*., 2012; Potvin *et al*., 2012). For example, the angle of approach and speed of lunging allow rorquals to minimize prey escape (Cade *et al*., 2020). Prey-capture success rate has been explicitly (Goldbogen *et al*., 2011; Guilpin *et al*., 2019; Guilpin *et al*., 2020) or implicitly (Wiedenmann *et al*., 2011; Pirotta *et al*., 2018b; Pirotta *et al*., 2019; Pirotta *et al*., 2021) assumed to be 100% in bioenergetic models for rorquals whales.

### The importance of prey

Estimates of time spent foraging or the number of successful foraging attempts are most useful in bioenergetics when combined with resource availability and quality estimates. In the past, studies have expressed energy intake requirements in weight of prey or as a percentage of body mass (e.g. Perez *et al*., 1990; Kastelein *et al*., 1997; Rosen and Worthy, 2018). However, as the energetic quality of prey items varies significantly with prey type and size, as well as in time and space, a more nuanced exploration of these factors is required.

A number of reviews exist that explore the diet of different marine mammal species and highlight the significant overlap between cetacean and pinniped diet (Tollit *et al*., 1997; Santos *et al*., 2001; Skern-Mauritzen *et al*., 2011; Andreasen *et al*., 2017; West *et al*., 2017; Trites and Spitz, 2018; Wilson and Hammond, 2019). However, many reviews on cetacean diet rely on stomach content samples that come from bycaught or stranded individuals, which may therefore provide a biassed assessment. Reviews of diet reveal that the composition varies by age, sex, region, season and inter-annually. Species fall on a generalist-specialist continuum (Jiang and Morin, 2005), though intra-population variation may exist (such that a generalist population may actually be composed of individual specialists) (Bolnick *et al*., 2003; Araújo *et al*., 2008; Araújo *et al*., 2011). Understanding where marine mammal species or individual are positioned on this continuum, and thus their plasticity in target habitat or prey, is important to understanding their resilience to disturbance (Gill *et al*., 2001; Booth, 2020; Hanson *et al*., 2021).

Generally, marine mammal diet is high in lipids and proteins and low in carbohydrates. While energy density is a useful metric when considering energetic balance, different energy metabolism pathways may exist, meaning different priorities for various micronutrients (Derous *et al*., 2021). Marine mammal metabolism may have evolved in response to a glucose-poor diet (a key difference from terrestrial mammals) and different macronutrients may have essential roles in metabolism, foraging behaviour and dive physiology (Derous *et al*., 2021).

A database was compiled using existing literature on prey type, size, nutritional content and availability (see Supplementary Information 1 and 2). A total of 146 literature sources were included, mainly consisting of peer-reviewed journal publications and publicly available grey literature. In some instances, species-level information was not available and prey data were presented at the family level only.

**Figure 2:**
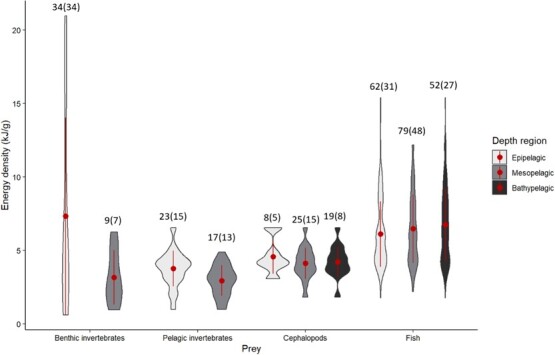
Violin plot for energy density of marine mamma l prey (wet weight) using information compiled in Supplementary Information 2. The red dot is the mean, lines are +/- 1 standard deviation. Numbers indicate the number of species (with families in p arentheses) for which energy density values were sourced.

**Figure 3:**
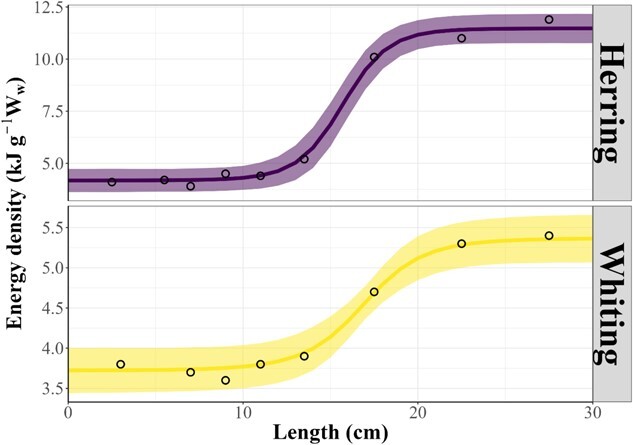
Predicted energy density (kJ g-1Ww) as a function of fish length using reported values from Pedersen and Hislop (2001) from Ju ly-September to fit a logistic model. Data in open circles, predicted mean in solid line, and confiden ce intervals displayed as ribbons per size class for herring and whiting.

Weight–length relationships were collated for 42 families and 78 species; the majority of these were fish species. Because mass is a cubic function of length in fish (Froese, 2006) and cephalopods (e.g. Dawe, 1988; Brunetti and Ivanovic, 1997) and the caloric value of a prey item is a product of mass, larger prey offer substantially greater energy gains (see Figure 1 of Booth, 2020). Energy density values were sourced for 114 families comprising 172 species, with many records from outside the marine mammal literature (Figure 2). Cephalopod species were consistently between 4 and 5 kJ g^−1^W_w_, irrespective of the water depth they inhabit, with estimates available for a moderate number of meso- and bathypelagic prey. Energy density of fish species was much more variable than for most other taxa. Other pelagic invertebrates generally had the lowest energy density, but a wide range of energy density of benthic invertebrates was found. Most are also typically small in size, yielding low total energy per item (Born *et al*., 2003). Marine mammals are also prey for some species (e.g. Kryukova *et al*., 2012; Pistorius *et al*., 2012; van Neer *et al*., 2015) and energy densities range between 4.6 and 5.1 kJ g^−1^W_w_ and 23 and34 kJ g^−1^W_w_ for muscle and blubber, respectively (Kuhnlein *et al*., 2002). Energy density varies within prey species, depending on length, sex and season (Hislop *et al*., 1991; Pierce *et al*., 1991; Pedersen and Hislop, 2001) (Figure 3). Macronutrient content in different prey types was available for only 22% of energy density records, suggesting this is a knowledge gap in the marine mammal field. However, understanding fish and cephalopod macronutrient composition is a burgeoning field of human-fisheries science (e.g. Hicks *et al*., 2019). Both weight–length relationships and energy densities were available for 34 families and 26 species, which could be used to estimate the energy density of observed prey species and size (e.g. from dietary studies).

### Prey density and availability

An organism’s fitness is determined by the relationship it has with its environment (i.e. resources, risks etc.) (Matthiopoulos *et al*., 2020). Consequently, the population size and individual fitness of a predator is linked to prey availability (MacArthur and Pianka, 1966; Trites and Donnelly, 2003; Øigård *et al*., 2013; Benoit-Bird, 2017; Benoit-Bird *et al*., 2020), prey quality (Österblom *et al*., 2008) and catchability (Brown *et al*., 1999; Balme *et al*., 2007). Of course, animals can partly compensate for reduced food availability depending on their ability to, for example, move to different foraging areas, alter their diet, reduce their metabolic costs or use stored energy. However, prey density is a key parameter for affecting marine mammal intake. We collated prey density information from 32 peer-reviewed marine mammal publications and a further 15 papers from outside the marine mammal literature (e.g. fishery data; Sala, 2018) (summarized in Supplementary Information 2).

Increasingly, prey density estimates are incorporated into marine mammal studies to understand spatio-temporal distribution patterns, predator–prey interactions, foraging ecology and costs of disturbance (Friedlaender *et al*., 2016; Stäbler *et al*., 2019; Guilpin *et al*., 2020; Pirotta *et al*., 2021). However, estimating prey availability to predators is difficult as it requires estimation of the spatio-temporal overlap between predator and prey species. Several approaches can be taken; some studies use proxies (Booth *et al*., 2013), average prey densities (Goldbogen *et al*., 2011), regional stock assessment estimates (Astarloa *et al*., 2021) or combine telemetry with fish survey data (Nowacek *et al*., 2011; Smout *et al*., 2014). Depending on the research question, large-scale stock trends may or may not be representative of food availability to a predator. Large-scale averages could help understanding broader spatial patterns, especially if prey distribution is persistent. However, the productivity of many fish species has undergone changes due to climate variability, commercial harvest, habitat degradation and/or alterations in competition (Brander, 2007; Baudron *et al*., 2020). Therefore, prey density estimates on a smaller spatio-temporal scale are required to increase understanding of foraging ecology and to quantify the consequences of anthropogenic disturbance. These could come from species distribution models (Smout *et al*., 2014; Pendleton *et al*., 2020), real-time monitoring (Friedlaender *et al*., 2006) or inter-prey spacing (Southall *et al*., 2019).

The varying diets of generalist marine mammals reflect changes in the availability of multiple prey species. Functional responses are crucial to understand trophic interactions and provide information on predation pressure, prey preference and population dynamics (Smout and Lindstrøm, 2007; Ransijn *et al*., 2021). To gain insight into diet adaptability, a Multi-Species Functional Response (MSFR) must be modelled. The MSFR describes how the consumption rate of a predator varies in relation to the availability of several prey species. Furthermore, it allows exploration of the consequences of future changes in prey-driven bottom-up processes, or the impact of top-down control on the rest of the ecosystem and the fisheries that depend on it.

### Food processing rates

While foraging, marine mammals make decisions affected by the rate at which they can acquire prey, which depends on the distribution and accessibility of prey and prey handling time (other factors such as predation, body condition, etc., may also factor into decision making). Additionally, the amount of food that an individual can consume is ultimately limited by digestive constraints, that is, the rate at which an animal can physically digest or process food (Williams *et al*., 2001; Rosen and Trites, 2004; Williams and Yeates, 2004). Food processing rates vary depending on the size and anatomy of the gastrointestinal tract, prey proximate composition (i.e. percent protein, lipid, and water) and prevalence of non-digestible structures (Trumble *et al*., 2003).

Few estimates of maximum food intake exist for marine mammals, but estimates are available from observations of animals in managed care (Goldblatt, 1993; Kastelein *et al*., 2019) or where a generalized relationship is assumed (sensu Taylor *et al*., 2007). Studies on juvenile Steller sea lions (*Eumetopias jubatus*) and Northern fur seals (*Callorhinus ursinus*) indicated that animals generally reached their digestive limit once food intake reached 14–32% of their body mass (Rosen and Trites, 2004; Rosen *et al*., 2012). This work highlighted that animals could alter their food intake in response to short-term changes in prey quality or availability, but that food intake levels could exceed their short-term physiological digestive capacities, impacting animal health (Kastelein *et al*., 2019). The wider taxonomic literature indicates that satiation levels can be impacted by numerous factors, including water intake, body weight and temperature (Reese and Hogenson, 1962; McFarland and L'Angellier, 1966; Arnason *et al*., 2009).

Rates of food ingestion are partly constrained by the rate at which animals can process it through the digestive system. This has been studied in several pinniped species. In harbour seals (*Phoca vitulina*), stomachs started to empty 1 h following feeding and some prey remained in the stomach after 5 h (Markussen, 1993). Kastelein *et al*. (2019) observed that porpoises had a large extensible forestomach (up to six times the relaxed size), are capable of ingesting > 90% of their daily energetic requirements (i.e. ~ 12–20 MJ) in 1 h and can feed again shortly afterwards. Most studies have measured processing time by measuring the time it takes for ingested chemical markers to appear in the faeces (Table 1). In general, passage rate is relatively uniformly rapid and among most studied pinniped species.

**Table 1:** Summary of empirically measured processing times (in hours) from marine mammal studies with associated prey type and markers used.

**Species**	**Marker**	**Prey**	**Processing time (hours)**	**Reference**
Pacific walrus	N:A.	unspecified fish	5–9	Fisher *et al*. (1992); Kastelein *et al*. (2003a)
Australian sea lion	T.O.	N.A.	6.5 (± 4.3)	Bodley *et al*. (1999)
New Zealand fur seal	T.O.	N.A.	4 (± 3)	Bodley *et al*. (1999)
Harbour seal	C	N.A.	6–14	Havinga (1933)
Harbour seal	C.R.D/B.S.	Fish	2.5–6.3	Markussen (1993)
Hawaiian monk seal	C.O.	Herring	14.0 (±4.8)	Goodman-Bacon (2018)
Bottlenose dolphin	C.R.D.	Herring and mackerel	3.9 (± 0.8)	Kastelein *et al*. (2003b)
False killer whale	C.R.D.	Herring and mackerel	3.9 (± 0.5)	Kastelein *et al*. (2000a)
Dusky dolphin	C.R.D.	Hake, squid, octopus, cuttlefish, misc. teleosts	2.5 (1.7–4.2)	Kastelein *et al*. (2000b)
Harbour porpoise	C.R.D.	Herring and sprat	2.4–3.3	Kastelein *et al*. (1997)
Beluga whale	C.R.D.	Herring, smelt, mackerel	4.5	Kastelein *et al*. (1994)
Amazon river dolphin	C.R.D.	Trout, carp, tench	4.2	Kastelein *et al*. (1999)
Manatee	N.A.	Water hyacinth	146	Lomolino and Ewel (1984)

SD or range is reported when available. N.A. data non available, T.O.—Titanium oxide, C—charcoal, C.R.D.—Carmine red dye, B.S.—Barium sulphate, C.O.—Chromic oxide

Little is known about the rate at which cetaceans process food. Most cetacean species have a forestomach, except beaked whales (Ziphiidae), Franciscana dolphin (*Pontoporia blainvillei*) and Baiji (*Lipotes vexillifer*) (Tarpley *et al*., 1987; Mead, 2009). Processing time for ingested food was estimated to ~ 14–15 h in common dolphins (*Delphinus delphis*) (Tomilin and Heptner, 1967), 2.5 h in harbour porpoises (Kastelein *et al*., 1997) and 3.6–4.5 h in bottlenose dolphins (*Tursiops truncatus*), dusky dolphins (*Sagmatias obscurus*), false killer whales (*Pseudorca crassidens*) and beluga whales (*Delphinapterus leucas*) (Kastelein *et al*, 1994, 2000ab, 2003b) (Table 1).

For baleen whales and balaenopterids in particular, the first volumetric estimate of forestomach capacity comes from Víkingsson (1997) for fin whales caught off Iceland. The forestomach volumetric capacity was estimated by either filling up the forestomach with water and subsequently measuring the volume, or estimating volume from natural gas expansion (Víkingsson, 1997). The author estimated a digestion time of ~15 h between the forestomach and the rectum and a clearance rate of the forestomach of ~3 h by establishing the relationship between length *L* (in m) and size of the forestomach *S* (in kg) (*S* = 0.47 *L*^2.36^) (Víkingsson, 1997). Wiedenmann *et al*. (2011) estimated a forestomach’s clearance rate of 4 h for blue whales using the equation of Víkingsson (1997). They then defined the rate at which the forestomach is filled to depend on forestomach capacity, swarm density and engulfment volume of a lunge. This food processing rate was used in bioenergetic models for blue whales (Wiedenmann *et al*., 2011; Pirotta *et al*., 2018b, 2019). In balaenid species, the rates of filling and clearing the forestomach might be different for rorquals, for which the forestomach accounts for a larger part of the total stomach volume (e.g. Tarpley *et al*., 1987; Víkingsson, 1997), but they have not been measured or modelled to date.

## **From IE to** NE

**Figure 4:**
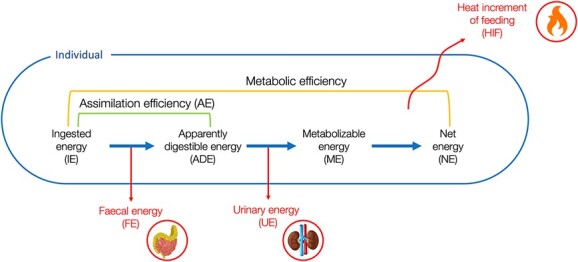
Detailed pathway from ingested energy (IE) to net energy (NE) and representation of associated losses and efficiencies.

Not all chemical energy ingested as food (gross energy intake or IE) is available to the animal to fuel its biological functions. The difference between IE and the resulting NE is due to several losses along the digestive process (Figure 4). Once ingested, the energy remaining after digestion and loss of FE is called apparently digested energy (ADE). Metabolizable energy (ME) is the remaining energy after subtraction of the UE from the ADE, that is, the energy lost as urea and other compounds in the urine. According to traditional bioenergetic schemes (e.g. Kleiber, 1975; Lavigne *et al*., 1982), the energy lost via the HIF resulting from digestive processes is subtracted from ME, leaving NE. The NE can be divided into energy available for growth, reproduction and storage, also termed production energy, and maintenance energy, which is the energy used to fuel other metabolic processes. The current state of knowledge of each parameter is reviewed below. While the emphasis of this review was on cetaceans, the literature on pinnipeds was reviewed where data for cetaceans were limited.

### Assimilation efficiency and FE

The efficiency with which an individual processes food can be differentiated between assimilation efficiency (AE) and metabolic efficiency (Figure 4).

To calculate AE, both the IE and energy lost through faecal material must be known. These two values are used to calculate the ADE:

$$ \begin{align*} &\mathrm{Assimilation}\ \mathrm{efficiency}\ \left(\mathrm{AE}\right)\notag\\&\quad=\frac{\mathrm{ingested}\ \mathrm{energy}\ \left(\mathrm{IE}\right)-\mathrm{faecal}\ \mathrm{energy}\ \left(\mathrm{FE}\right)\ }{\mathrm{ingested}\ \mathrm{energy}\ \left(\mathrm{IE}\right)}\\
&\quad=\frac{\mathrm{apparent}\ \mathrm{digestible}\ \mathrm{energy}\ \left(\mathrm{ADE}\right)\ }{\mathrm{ingested}\ \mathrm{energy}\ \left(\mathrm{IE}\right)} \end{align*}$$

AE is also referred to as digestive efficiency (Rosen and Trites, 2000) or apparent digestibility (Costa and Williams, 1999). However, some studies use these terms when they are actually reporting dry matter digestibility (Lawson *et al*., 1997a; Rosen and Trites, 2000) and dry matter disappearance (Nordøy *et al*., 1993), which are measures of the ratio of lost dry organic matter, rather than energy per se. Dry matter digestibility is usually lower than AE (Fadely *et al*., 1994); while similar, the two measures are not equivalent (Rosen and Trites, 2000). In some literature, including some studies on cetaceans (Fortune *et al*., 2013) and other taxa (i.e. reptiles, McConnachie and Alexander, 2004), the term AE is used to account for both faecal and UE losses, but this is more correctly termed metabolic efficiency. Metabolic efficiency is a measure of how efficient an individual is at processing food and covers both urinary and FE losses, resulting in ME (Lavigne *et al*., 1982; Rosen and Worthy, 2018).

As AE’s quantification relies on the analysis of faecal material, it has been mostly measured in pinnipeds for logistical reasons (Table 2). In general, AE for pinnipeds is high, particularly for fish prey species compared with invertebrates. AE is affected by the biochemical composition of the prey (Schneider and Flatt, 1975; Lawson *et al*., 1997a; Rosen and Trites, 2000) but does not seem to be affected by meal size or frequency of feeding (Keiver *et al*., 1984; Ronald *et al*., 1984; Lawson *et al*., 1997a; Lawson *et al*., 1997b; Rosen *et al*., 2000). In experiments using fish species differing in energy density and lipid/protein content, AE was higher for prey species with a higher energy and lipid content than those with lower energy, higher protein content (Lawson *et al*., 1997a, 1997b; Rosen *et al*., 2000; Rosen and Trites, 2000; Diaz Gomez *et al*., 2016). A study with northern fur seals demonstrated that AE was negatively related to protein content, possibly because proteins decrease lipid digestibility (Diaz Gomez *et al*., 2020).

**Table 2:** Summary of values measured or used for marine mammal species for both AE (%) representing the fraction of IE retained after FE losses, and FE loss (expressed as a % of IE) is the amount of energy lost in the faecal matter.

**Species**	**Parameter**	**Value**	**Unit**	**Prey**	**Reference**
Bowhead whale	AE	90	%	Calanoids	Laidre *et al*. (2007) ^**^
Baleen whales	AE	80	%	N.A.	Lockyer (1981) ^**^
Minke whale	AE	92.1 83.4	%	Herring Krill	Nordøy *et al*. (1993) ^*^
Minke whale	AE	87–93	%	Krill	Mårtensson *et al*. (1994a) ^*^
Crabeater seal		84.0	%	Krill	Mårtensson *et al*. (1994a) ^*^
Grey seal	AE	92.6	%	Herring	Ronald *et al*. (1984) ^*^
Grey seal	AE	92.8	%	Mixed diet	Prime and Hammond (1987) ^*^
Harbour seal	AE	92.0 88.5		Herring pollock	Trumble *et al*. (2003) ^*^
Harp seal	AE	72.2 92.5–95	%	Shrimp herring	Keiver *et al*. (1984) ^*^
Harp seal	AE	91.0 95.0 84.3	%	Capelin Herring Atlantic cod	Lawson *et al*. (1997b) ^*^
Harp seal	AE	93–94 81–83	%	Capelin Invertebrates	Mårtensson *et al*. (1994b) ^*^
Ringed seal	AE	97.0	%	Herring	Parsons (1977) ^*^
Ringed seal	AE	92.0–95.0 72.2	%	Herring Shrimp	Keiver *et al*. (1984) ^*^
Ringed seal	AE	83.2 86.6 88.3 93.8 82.1	%	Red fish Capelin Cod Herring Herring/shrimp	Lawson *et al*. (1997a) ^*^
Walrus	AE	92.7	%	Herring clam	Fisher *et al*. (1992) ^*^
Steller sea lion	AE	95.4 93.9 90.4 93.4	%	Herring Pollock Squid Salmon	Rosen and Trites (2000) ^*^
Northern fur seal	AE	96.0 96.9 96.3 95.9–96.7	%	Capelin Herring Pollock Mixed prey diets	Diaz Gomez *et al*. (2016) ^*^
Northern fur seal	AE	90.0	%	Fish	Fadely *et al*. (1990) ^*^
Californian sea lion	AE	88.0–91.0 83.0–91.0	%	Herring Pollock	Fadely *et al*. (1994) †
West Indian manatee	AE	80.0–88.8		Water hyacinths, lettuce	Lomolino and Ewel (1984) ^*^
North Atlantic right whale	FE	6	% of IE	Copepods	Fortune *et al*. (2013) ^**^
Minke whale	FE	~8	% of IE	na	Folkow *et al*. (2000) ^**^
Grey whale	FE	20	% of IE	na	Greenwald (2005) ^**^
Sea otter	FE	18	% of IE	na	Costa (1982) ^*^

Symbols associated with references indicate the methodology used for the values listed: ^*^ from experiment, ^**^ assumed or † − original reference not available. N.A. data not available

Differences in AE between fish species with different proximate composition are relatively minor compared to differences between fish and invertebrate prey. For example, several studies on captive harp seals (*Phoca groenlandica*) found that AE was higher when seals were fed fish (92.5–97.0%) compared with small crustaceans like krill (Family *Euphausiidae*) (81–83%; Mårtensson *et al*., 1994b) or shrimp (Family *Caridea*) (72.2%; Keiver *et al*., 1984). A study with crabeater seals (*Lobodon carcinophagus*) reported a similarly low AE (84.0%) for krill (Mårtensson *et al*., 1994a). No such difference in AE was reported with walruses (*Odobenus rosmarus*) that were fed herring (*Clupea harengus*) versus clams (*Mercenaria mercenaria*) (92.7%), even though the lipid content of herring was 23.5% higher than that of the clams (Fisher *et al*., 1992). AE was significantly higher in female (94.4%) than male (91%) walruses but was not correlated with age (Fisher *et al*., 1992).

The AE of baleen whales was first estimated at 80% (Lockyer, 1981), based on the assumption that this upper limit could not be exceeded because of the indigestible exoskeleton of chitinous prey, while also accounting for fish being in the diet of many baleen whales species. This estimate has been widely used in the literature (Kenney *et al*., 1986; Sigurjónsson and Víkingsson, 1997; Baumgartner *et al*., 2003; Laidre *et al*., 2007; Goldbogen *et al*., 2011; Wiedenmann *et al*., 2011; Braithwaite *et al*., 2015; Pirotta *et al*., 2018b; Guilpin *et al*., 2019; Pirotta *et al*., 2019; Guilpin *et al*., 2020; Pirotta *et al*., 2021). Digestive tracts and microbiomes of baleen whales have been studied for nearly 40 years (Herwig et al., 1984; Herwig and Staley, 1986; Tarpley et al., 1987; Mårtensson et al., 1994a; Olsen et al., 1994a; Olsen et al., 1994b; Haug et al., 1995; Mathiesen et al., 1995; Miller et al., 2020). More recently, it has been shown that baleen whales have specialized gut microbiome, such as chitinolytic bacteria, that allow them to digest chitin (e.g. the exoskeleton of euphausiids) and extract the nutrients therein (Olsen *et al*., 2000; Sanders *et al*., 2015), suggesting that 80% is an underestimate. AE may thus be closer to the 93% estimated for krill-eating minke whales (Mårtensson *et al*., 1994a) (estimated using dietary manganese as an inert marker).

### UE **loss**

UE is the chemical energy lost as urea and other metabolic end products in the urine. UE is represented as a percentage of the ADE and is proportional to the nitrogen content of prey items (Keiver *et al*., 1984; Worthy, 1990; Rosen and Worthy, 2018). That is, it is proportional to the nitrogen absorbed in the gut and not the nitrogen ingested (i.e. discounting the fraction lost through the faeces). UE was first assumed to be ~ 8% of the digestible energy (DE) based on a review of values from terrestrial mammals (Lavigne *et al*. (1982). Literature on UE for marine mammals is limited to pinnipeds, for which few measurements exist from feeding experiments of captive individuals, that is, for harp seal, grey seal (*Halichoerus grypus*) and ringed seal (*Phoca hispida*) (Table 3). Feeding experiments vary in prey type (which differ in biochemical composition) and meal size. In one of the most thorough studies, Keiver *et al*. (1984) analysed urine samples for energy and nitrogen content, urea, creatinine and uric acid. They found that UE was strongly dependent on the apparent digestible nitrogen intake, allowing predictions of UE given measures of AE and/or prey proximate composition (e.g. Diaz Gomez *et al*., 2016)).

**Table 3:** UE losses measured or used for marine mammal species, expressed as either a percentage of the IE or a percentage of the ADE, which accounts for FE losses.

**Species**	UE **loss**	**Unit**	**Reference**
Sea otter	10	% of IE	Costa (1982) ^*^
North Atlantic right whale	8	% of IE	Fortune *et al*. (2013) ^**^
Grey seal	7.9	% of ADE	Ronald *et al*. (1984) ^*^
Ringed seals	8.6	% of ADE	Parsons (1977) †
Pinnipeds	8	% of ADE	Lavigne *et al*. (1982) ^**^
Grey whale	10	% of ADE	Greenwald (2005) ^**^
Harp seal	6.5–9.5	% of ADE	Keiver *et al*. (1984) ^*^
Minke whale	8	% of ADE	Folkow *et al*. (2000) ^**^

Symbols associated with references indicate the methodology used for the values listed: ^*^ from experiment, ^**^ assumed or † − original reference not available

For cetaceans, information on the proportion of energy lost through urine is limited and no estimates of UE exist. In bioenergetic studies, UE has either been overlooked, taken from measurements and estimates from pinnipeds or terrestrial taxa (Lavigne *et al*., 1982), or estimated based on the nitrogen content of prey (Fortune *et al*., 2013) (Table 3).

### Heat increment of feeding

The HIF, also referred to as specific dynamic action (SDA), is a postprandial obligatory metabolic mechanism. It represents the increase in metabolic rate resulting from the physical and biochemical processes of digestion (preabsorptive, absorptive and post-absorptive) (Brody, 1945). The physiological processes underpinning the HIF are numerous, complex and non-exhaustively described in McCue (2006). The HIF can account for a substantial portion of IE and should ideally be included as a separate parameter in bioenergetic models. Nevertheless, this is not always possible, as the costs of HIF are incorporated in metabolic rates estimated from doubly labelled water. Given the scarcity of values for this parameter for marine mammals, and cetaceans in particular, most studies have not explicitly taken these costs into account (Wiedenmann *et al*., 2011; Pirotta *et al*., 2018b; Pirotta *et al*., 2019; Guilpin *et al*., 2020). Indeed, digestion costs are oftentimes assumed to be included in estimates of field metabolic rate (Blix and Folkow, 1995; Nordøy *et al*., 1995; McHuron *et al*., 2020). The HIF has been explicitly accounted for in a small number of bioenergetic studies of large whales, based on estimates from the pinniped literature, for example, grey whale or North Atlantic right whale (Greenwald, 2005; Fortune *et al*., 2013).

The HIF depends on the size and composition of the meal (Hoch, 1971), and the age and nutritional state of the animal (Brody, 1945). The chemical composition of the meal affects total HIF, given that the digestion of carbohydrates, proteins or lipids increases metabolism differently in amplitude and duration (Blaxter, 1989). The cost of processing carbohydrates has been estimated to be 6% of the IE, 13% of IE for processing fat and up to 30% of IE when processing protein (Bartholomew, 1977). The duration of an increase in metabolism linked to the HIF has been empirically estimated to 5 h for carbohydrates, 9 h for lipid and 12 h for protein (Hoch, 1971; Worthy, 1990). Consistent with other vertebrates, both the total increase in metabolism and the duration of the effect in marine mammals have been shown to depend on diet composition and meal size (Rosen and Trites, 1997; Costa and Williams, 1999; Costa, 2009). Unfortunately, the HIF cannot be calculated directly from diet composition, as the mixed composition of food items results in a lower than predicted HIF (Hoch, 1971).

Although accurate estimates are needed for bioenergetic models, measurements of the amplitude and duration of HIF are only possible for captive animals, for which fasting, meal size and composition can be controlled and monitored. The HIF is empirically measured by quantifying the increase in metabolism (measured as rate of oxygen consumption) over several hours following a meal of known size and composition. The HIF has been measured in few species: sea otter, harp seal, harbour seal, ringed seals, northern elephant seals (*Mirounga angustirostris)*, Steller sea lions and northern fur seals (Table 4).

**Table 4:** Summary of measured HIF values (% IE and ± SD, when available) and associated characteristics in marine mammal species, specifically pinnipeds and mustelids.

**Species**	**HIF (% IE)**	**Duration of HIF**	**Prey**	**Reference**
Sea otter	13.2 ± 1.4SD	4-5 h	Squid	Costa and Kooyman (1984) ^*^
	10	4-5 h	Clam	
Steller sea lion	9.9 ± 0.9 (small meal) 12.4 ± 0.9 (large meal)	6-8 h (small meal) 8-10 h (large meal)	Herring + other unspecified fish species	Rosen and Trites (1997) ^*^
Harp seal	17	5-6 h	Herring	Gallivan and Ronald (1981) ^*^
Harbour seal	14.9	Max 12 h	Herring	Markussen *et al*. (1994) ^*^
	4.7 5.7	10 h	Herring Pollock	Ashwell-Erickson (1981) ^*^
Ringed seals	27–35% increase in metabolism over RMR	12-13 h and peak after 4-6 h	N.A.	Parsons (1977) ^*^
Northern fur seals	4.3 ± 1.0 6.5 ± 3.8 12.4 ± 2.0 7.1 ± 2.3 7.9 ± 3.0 6.0 ± 1.5 6.9 ± 2.0 5.2 ± 1.1	N.A.	Pacific herring walleye pollock capelin herring + pollock herring + capelin herring + magister armhook squid pollock + capelin herring + pollock + capelin	Diaz Gomez *et al*. (2016) ^*^
South American fur seals	61% increase in metabolism over RMR	N.A.	white croaker + striped weakfish + Brazilian menhaden	Dassis *et al*. (2014) ^*^
Northern elephant seal	9.1–11.4 11.5–13.0	N.A.	herring capelin	Barbour (1993) ^*^

Symbols associated with references indicate the methodology used to estimate the values listed: * from experiment. N.A. data non available

Although generally considered a waste product in most bioenergetic models, there are cases where the HIF can be repurposed (Rosen *et al*., 2007). For endothermic animals, the heat produced during digestion could be used to offset costs associated with thermoregulation (Costa and Kooyman, 1984), in a process termed thermal substitution. This hypothesis is difficult to verify and quantify as its effect would likely depend upon multiple factors, including the temperature of the environment and the nutritional state and body condition of the individual. Thermal substitution with HIF has been demonstrated in sea otters (Costa and Kooyman, 1984). However, it should be noted that this species is very different from other marine mammals, such that they rely on fur for thermoregulation while inhabiting cold environments. In contrast, thermal substitution with HIF did not occur in the much larger Steller sea lion (Rosen and Trites, 2003), another species which inhabits cold environments.

## Discussion/Conclusions

The focus in bioenergetic modelling is often on the energetic costs an animal incurs, but the robust estimation of an individual’s energy intake is equally critical for producing meaningful predictions. We have reviewed the components and processes that affect energy intake from ingested gross energy to biologically useful units of NE. Processes that determine energy intake can be conceptually separated into sets (Figure 1) with some parameters contributing to the estimation of IE and other parameters associated with digestive processes.

The study of marine mammal foraging effort has tremendously benefitted from the ever-advancing field of biologging. Biologging devices that allow the measurement of foraging effort at different temporal and spatial scales exist or are being developed (Williams and Ponganis, 2021). Furthermore, advances in tag technology and analytical methods make it possible to monitor energy intake and health metrics like body condition regularly and across a large number of individuals (Arce *et al*., 2019; Hooker *et al*., 2019; Aoki *et al*., 2021; Siegal *et al*., 2022). Limitations would then be more associated with the costs of such studies and the logistical challenges of deploying biologgers. Regarding prey, our review showed that valuable data on prey distribution, behaviour, biomass, density, energy content and composition exist in the literature. This is an area that is being advanced with novel prey monitoring techniques, for example, autonomous underwater vehicles with autonomous echosounder systems or environmental DNA (eDNA) (Southall *et al*., 2019; Benoit-Bird *et al*., 2020; Urmy and Benoit-Bird, 2021; Visser *et al*., 2021). Prey-capture success rates remain relatively uncertain, but estimates for pinnipeds, odontocetes and mysticetes are increasingly available from biologgers (Kuhn and Costa, 2006; Wisniewska *et al*., 2016; Cade *et al*., 2020).

Once energy is ingested, it goes through the digestive process and associated energy losses, resulting in NE available to the individual for maintenance and production. The energy losses associated with faecal matter, urine production and the HIF are difficult to measure in free-ranging marine mammals, especially cetaceans. Most of the parameters used in cetaceans bioenergetic studies (Greenwald, 2005; Wiedenmann *et al*., 2011; Fortune *et al*., 2013; Villegas-Amtmann *et al*. 2015; Pirotta *et al*., 2018a) are either modelled or scaled from terrestrial mammal species or empirically measured from pinnipeds and mustelids. While adapting pinniped estimates to cetaceans could provide a first step, some parameters cannot be applied across all taxonomic groups. For instance, estimating UE loss based on known estimates from pinnipeds, adjusted based on the biochemical composition of cetacean prey, can provide an interim solution (Fortune *et al*., 2013). In contrast, applying an estimate of HIF from mustelids to cetaceans might not be appropriate considering their highly different physiology. The accurate estimation of these parameters represents the largest knowledge gap when quantifying energy intake, for cetaceans in particular.

With the ultimate goal of improving bioenergetic modelling, this review highlights the current empirical information on important parameters, which can be utilized in the latest modelling approaches (Pirotta, 2022, this Special Issue) to collectively drive this research topic ahead and improve conservation efforts for impacted species and populations. Sensitivity analyses of available models, which now span the reproductive strategies of most marine mammals, would be very useful to help identify uncertain and impactful parameters and guide research effort.

Animals achieving energetic balance are a key to their reproduction and survival (Costa and Williams, 1999; Parsons, 2005). As climate change is affecting terrestrial and marine ecosystems, understanding how the energetic landscape is being impacted (e.g. via changes in prey composition, size distribution or energetic content), how different species are responding and robustly projecting how this will propagate in the future (e.g. Gallagher *et al*. 2021b) remains critical to inform conservation and management.

## Funding

This work was primarily funded under an award from Office of Naval Research: N000142012392, and with support from the Marine Mammal Commission project: “*A priority setting exercise to identify key unanswered questions in marine mammal bioenergetics”*. Funding from the Joint Nature Conservation Committee supported fish energy analyses - award C18-0241-1285.

## Data Availability

All data are incorporated into the article and its online supplementary material.

## Supplementary Material

Web_Material_coac083
