# Supplementary material for: Estimating energetic intake for marine mammal bioenergetic models
Source: Conserv Physiol. 2023 Feb 4;11(1):coac083. doi: 10.1093/conphys/coac083 (PMC9900471; doi:10.1093/conphys/coac083)
Supplement: Web_Material_coac083 [file web_material_coac083.zip › EnergyIntake_SuppInfo1.docx]

**Estimating energetic intake of marine mammal in bioenergetic models – Supplementary Information 1.**

**Review of marine mammal prey**

This repository holds the data collated as part of this review. The review of prey and structure of the data present is skewed heavily towards to cetaceans as this was the original focus of the review. However, the review identified significant overlap in the prey items of cetaceans and pinnipeds. Each of the sheets are filterable to allow ease of access.

There are seven excel sheets containing information on marine mammal prey:

- Prey type – summarising the prey species that different marine mammals feed on.
- Prey size – the observed/estimated size of marine mammal prey
- W-L relationships: Weight-Length relationships sourced for a variety of prey species
- Prey_ED - energy density values for known marine mammal prey species. Prey species were grouped into fish species, cephalopod species and benthic and (other) pelagic invertebrates. Highlighted rows indicate energy density (kJ/g) values that have been converted from dry weight values to wet weight values where data on moisture content (%) was available (for production of Figure 2 of manuscript).
- Density_Fish – density estimate available for fish species in marine mammal diet. (See Notes for highlighted cells – flagging uncertainty in their interpretation.)
- Density_Cephalopod – density estimate available for cephalopod species in marine mammal diet.
- Density_Other – density estimate available for non-fish and non-cephalopod marine mammal prey species

The following conversion was applied to estimate wet weight from dry weight values available in the literature:

$$W_{w}= W_{d}\left( \frac{100-M}{100} \right)$$

Where W_w_ is the energy density (kJ g^-1^) for wet weight samples, W_d_ is the energy density (kJ g^-1^) for dry weight samples and M is the percentage of moisture content present in the sample.

A number of dry weight energy density values were not converted due to a lack of moisture content for values in the literature.

**Prediction of fish energy density with length (Figure 4)**

Published estimates (Pederson & Hislop 2001) by size class in the North Sea during quarter 3 (July-September) were used to predict energy density values (kJ g^-1^W_w_) for the two fish species (*i.e.* herring and whiting) from a fitted non-linear logistic model. The model was fitted using non-linear least-squares techniques performed with the nls function from the R package “stats”.

The logistic functions can be expressed as the following equations:

$$lp=a+b*l$$

$$E=c+s \frac{{lp}^{2}}{1+{lp}^{2}}$$

where lp is the linear predictor, l is the length of the fish in cm, E is the energy density value, c represents the intercept, s is the scale that 'stretches' it from the original (0,1), a and b are logistic parameters.

Reported values are represented in Figure 4 by open circles, model predictions by solid lines and the ribbons represents the associated confidence intervals. The correlation between predictions and the data was 99.6% for both herring and whiting indicating a very good fit of the model. The variances of the model were similar for both species but slightly higher for herring.

**Table references**

Literature cited in Tables 1-4 are listed below:

Ashwell-Erickson, S. M. 1981. The energy cost of free existence for Bering Sea harbor and spotted seals. University of Alaska Fairbanks.

Barbour, A. S. 1993. Heat increment of feeding in juvenile northern elephant seals. University of California, Santa Cruz.

Bodley, B., and M. Bryden. 1999. Rate of passage of digesta through the alimentary tract of the New Zealand fur seal (Arctocephalus forsteri) and the Australian sea lion (Neophoca cinerea)(Carnivora: Otariidae). australian Journal of Zoology **47**:193-198.

Costa, D. P. 1982. Energy, Nitrogen, and Electrolyte Flux and Sea Water Drinking in the Sea Otter Enhydra Lutris. Physiological Zoology **55**:35-44.

Costa, D. P., and G. L. Kooyman. 1984. Contribution of specific dynamic action to heat balance and thermoregulation in the sea otter Enhydra lutris. Physiological Zoology **57**:199-203.

Dassis, M., D. H. Rodriguez, E. Ieno, P. E. Denuncio, J. Loureiro, and R. Davis. 2014. Resting metabolic rate and heat increment of feeding in juvenile South American fur seals (Arctocephalus australis). Comparative Biochemistry and Physiology Part A: Molecular & Integrative Physiology **168**:63-68.

Diaz Gomez, M., D. A. Rosen, and A. W. Trites. 2016. Net energy gained by northern fur seals (Callorhinus ursinus) is impacted more by diet quality than by diet diversity. Canadian Journal of Zoology **94**:123-135.

Fadely, B., J. Zeligs, and D. Costa. 1994. Assimilation efficiencies and maintenance requirements of California sea lions (Zalophus californianus) fed walleye pollock (Theragra chalcogramma) and herring (Clupea harengus). Final Report to the National Marine Mammal Laboratory, Alaska Fisheries Science Center, National Marine Fisheries Service **7600**:98115-90070.

Fadely, B. S., G. A. Worthy, and D. P. Costa. 1990. Assimilation efficiency of northern fur seals determined using dietary manganese. The Journal of Wildlife Management:246-251.

Fisher, K., R. Stewart, R. Kastelein, and L. Campbell. 1992. Apparent digestive efficiency in walruses (Odobenus rosmarus) fed herring (Clupea harengus) and clams (Spisula sp.). Canadian Journal of Zoology **70**:30-36.

Folkow, L. P., T. Haug, K. T. Nilssen, and E. S. Nordøy. 2000. Estimated food consumption of minke whales Balaenoptera acutorostrata in Northeast Atlantic waters in 1992-1995. Pages 65-80 NAMMCO Scientific Publications.

Fortune, S. M., A. W. Trites, C. A. Mayo, D. A. Rosen, and P. K. Hamilton. 2013. Energetic requirements of North Atlantic right whales and the implications for species recovery. Marine Ecology Progress Series **478**:253-272.

Gallivan, G., and K. Ronald. 1981. Apparent specific dynamic action in the harp seal (Phoca groenlandica). Comparative Biochemistry and Physiology Part A: Physiology **69**:579-581.

Greenwald, N. L. E. 2005. A Theoretical Approach To Assessing Annual Energy Balance In Gray Whales (eschrichtius Robustus).

Havinga, B. 1933. Der Seehund in den Holländischen Gewässern. . Tijdschri van de Nederlands Dierkundige Vereeniging **3**:79-111.

Kastelein, R., J. Ford, E. Berghout, P. Wiepkema, and M. Van Boxsel. 1994. Food consumption, growth and reproduction of belugas(Delphinapterus leucas) in human care. Aquatic Mammals **20**:81-97.

Kastelein, R., W. Klasen, J. Postma, H. Boer, and P. Wiepkema. 2003a. Food consumption, growth and food passage times in Pacific walrus. International Zoo Yearbook **38**:192-203.

Kastelein, R., J. Mosterd, N. Schooneman, and P. Wiepkema. 2000a. Food consumption, growth, body dimensions, and respiration rates of captive false killer whales (Pseudorca crassidens). Aquatic Mammals **26**:33-44.

Kastelein, R., C. Staal, and P. Wiepkema. 2003b. Food consumption, food passage time, and body measurements of captive Atlantic bottlenose dolphins (Tursiops truncatus). Aquatic Mammals **29**:53-66.

Kastelein, R., C. Van Der Elst, H. Tennant, and P. Wiepkema. 2000b. Food consumption and growth of a female dusky dolphin (Lagenorhynchus obscurus). Zoo Biology: Published in affiliation with the American Zoo and Aquarium Association **19**:131-142.

Kastelein, R. A., J. Hardeman, and H. Boer. 1997. Food consumption and body weight of harbour porpoises (Phocoena phocoena). The biology of harbour porpoise:217-233.

Keiver, K. M., K. Ronald, and F. W. H. Beamish. 1984. Metabolizable energy requirements for maintenance and faecal and urinary losses of juvenile harp seals (Phoca groenlandica). Canadian Journal of Zoology **62**:769-776.

Laidre, K. L., M. P. Heide, and T. G. Nielsen. 2007. Role of the bowhead whale as a predator in West Greenland. Marine Ecology Progress Series **346**:285-297.

Lavigne, D., W. Barchard, S. Innes, and N. Øritsland. 1982. Pinniped bioenergetics. Mammals in the seas **4**:191-235.

Lawson, J. W., J. A. Hare, E. Noseworthy, and J. K. Friel. 1997a. Assimilation efficiency of captive ringed seals (Phoca hispida) fed different diets. Polar Biology **18**:107-111.

Lawson, J. W., E. H. Miller, and E. Noseworthy. 1997b. Variation in assimilation efficiency and digestive efficiency of captive harp seals (Phoca groenlandica).

Lockyer, C. 1981. Growth and energy budgets of large baleen whales from the southern hemisphere.

Lomolino, M. V., and K. C. Ewel. 1984. Digestive efficiencies of the West Indian manatee (Trichechus manatus). Florida Scientist:176-179.

Markussen, N. H. 1993. Transit time of digesta in captive harbour seals ( Phoca vitulina ). Pages 1071-1073 Canadian Journal of Zoology.

Markussen, N. H., M. Ryg, and N. A. Øritsland. 1994. The effect of feeding on the metabolic rate in harbour seals (Phoca vitulina). Pages 89-93 Journal of Comparative Physiology B.

Mårtensson, P.-E., E. Nordøy, and A. Blix. 1994a. Digestibility of krill (Euphausia superba and Thysanoessa sp.) in minke whales (Balaenoptera acutorostrata) and crabeater seals (Lobodon carcinophagus). British Journal of Nutrition **72**:713-716.

Mårtensson, P. E., E. Nordøy, and A. Blix. 1994b. Digestibility of crustaceans and capelin in harp seals (Phoca groenlandica). Marine Mammal Science **10**:325-331.

Nordøy, E. S., W. Sørmo, and A. S. Blix. 1993. In vitro digestibility of different prey species of minke whales (Balaenoptera acutorostrata). British Journal of Nutrition **70**:485-489.

Parsons, J. L. 1977. Metabolic studies in ringed seals (Phoca hispida). University of Guelph.

Prime, J. H., and P. S. Hammond. 1987. Quantitative assessment of grey seal diet from faecal analysis.*in* A. C. Huntley, D. P. Costa, G. A. J. Worthy, and M. A. Castellini, editors. Approaches to marine mammal energetics. Allen Press, Lawrence, KS 66044, USA.

Ronald, K., K. M. Keiver, F. W. H. Beamish, and R. Frank. 1984. Energy requirements for maintenance and faecal and urinary losses of the grey seal (Halichoerus grypus). Canadian Journal of Zoology **62**:1101-1105.

Rosen, D. A., and A. Trites. 2000. Digestive efficiency and dry-matter digestibility in Steller sea lions fed herring, pollock, squid, and salmon. Canadian Journal of Zoology **78**:234-239.

Rosen, D. A. S., and A. W. Trites. 1997. Heat increment of feeding in Steller sea lions, Eumetopias jubatus. Pages 877-881 Comparative Biochemistry and Physiology Part A: Physiology.

Trumble, S., P. Barboza, and M. Castellini. 2003. Digestive constraints on an aquatic carnivore: effects of feeding frequency and prey composition on harbor seals. Journal of Comparative Physiology B **173**:501-509.
